# One Year Follow-Up of Taste-Related Reward Associations with Weight Loss Suggests a Critical Time to Mitigate Weight Regain Following Bariatric Surgery

**DOI:** 10.3390/nu13113943

**Published:** 2021-11-04

**Authors:** Kimberly R. Smith, Anahys Aghababian, Afroditi Papantoni, Maria G. Veldhuizen, Vidyulata Kamath, Civonnia Harris, Timothy H. Moran, Susan Carnell, Kimberley E. Steele

**Affiliations:** 1 Department of Psychiatry & Behavioral Sciences, Johns Hopkins University School of Medicine, Baltimore, MD 21205, USA; kimberly.smith@jhmi.edu (K.R.S.); tmoran@jhmi.edu (T.H.M.); 2 Division of Child and Adolescent Psychiatry, Department of Psychiatry & Behavioral Sciences, Johns Hopkins University School of Medicine, Baltimore, MD 21205, USA; aaghaba1@jhmi.edu (A.A.); afroditi@live.unc.edu (A.P.); susan.carnell@jhmi.edu (S.C.); 3 Anatomy Department, Mersin University School of Medicine, 32133 Mersin, Turkey; margaveldhuizen@gmail.com; 4 Division of Medical Psychology, Department of Psychiatry & Behavioral Sciences, Johns Hopkins University School of Medicine, Baltimore, MD 21205, USA; vkamath@jhmi.edu; 5 Johns Hopkins Center for Bariatric Surgery, Department of Surgery, Johns Hopkins University School of Medicine, Baltimore, MD 21205, USA; harris.civonnia@gmail.com

**Keywords:** bariatric surgery, Roux-en-Y gastric bypass, vertical sleeve gastrectomy, taste, reward, weight loss

## Abstract

Background: Weight regain is a concerning issue in bariatric patients. We previously demonstrated that taste-related reward processing was associated with six-month weight loss outcomes following Roux-en-Y gastric bypass (RYGB) but not vertical sleeve gastrectomy (VSG). Here, we assessed whether these taste factors persisted in predicting weight loss, and weight regain, at one year post-surgery. Methods: Adult women enrolled in a longitudinal study of taste preferences following bariatric surgery completed behavioral and neuroimaging assessments at one year post-surgery. Results: RYGB produced better weight loss relative to VSG, with weight regain and greater weight loss variability observed from six months to one year post-VSG. Changes in liking for high fat at 2 weeks post-surgery from baseline remained a predictor of weight loss in RYGB, but other predictors did not persist. Average liking ratings rebounded to baseline and higher self-reported food cravings and dietary disinhibition correlated with poorer weight loss at one year post-surgery. Conclusion: Initial anatomical and metabolic changes resulting from RYGB that reset neural processing of reward stimuli in the mesolimbic pathway appear to be temporary and may be contingent upon post-operative eating behaviors returning to preoperative obesogenic tendencies. Six months post-surgery may be a critical window for implementing interventions to mitigate weight gain.

## 1. Introduction

Data from bariatric patients suggest two phases of weight loss following bariatric surgery—the dynamic weight loss phase and the weight maintenance phase. During the dynamic phase, weight loss is rapid, with our previous study showing a ~20-pound decrease two weeks after surgery [1]. During the weight maintenance phase, rate of weight loss slows and a nadir is typically reached within 2 years of surgery [2] followed by an increase in risk for weight regain. The lack of a standardized definition for weight regain following bariatric surgery has hindered an accurate identification of the number of patients who experience weight regain, with reports ranging between 4 and 90% within the first five years of bariatric surgery [3,4,5,6]. Regardless, weight regain is a significant issue with adverse physical and psychological repercussions in bariatric patients.

Roux-en-Y gastric bypass (RYGB) and vertical sleeve gastrectomy (VSG) are the two most commonly performed bariatric procedures for the treatment of obesity and obesity-related comorbidities [7]. While both surgeries result in sustained weight loss relative to baseline body weight, RYGB has been shown to be effective in producing more rapid and greater weight loss compared with VSG [1]. The mechanisms responsible for the superiority of RYGB relative to VSG in weight reduction are unclear. In our recent work, we demonstrated postoperative neural and behavioral alterations that may underlie the observed improved weight loss outcomes in patients receiving RYGB. Specifically, preoperative liking ratings of sucrose-sweetened taste mixtures were positively associated with greater percent total weight loss (%TWL) in RYGB, but not VSG, and patients receiving RYGB who reported acute decreases in liking for high-fat mixtures lost more %TWL at six months than those who reported no change or an increase in liking for the high fat mixtures [1]. This aligns with previous studies demonstrating changes in taste processing, including decreases in reported sugar taste preferences [8,9,10] and increases in “sweet” taste sensitivity [9,11,12]. Further, patients who showed lower blood-oxygen-level-dependent (BOLD) responses in the ventral tegmental area (VTA), a brain reward region, as measured by functional magnetic resonance imaging (fMRI) to sucrose and fat mixtures prior to RYGB lost more weight six months post-surgery than RYGB recipients who exhibited higher VTA responses [1].

Here, one year post-operative behavioral and fMRI data in patients who were enrolled in a longitudinal study assessing taste preferences following bariatric surgery [1] were analyzed to determine if the taste-related predictors of six-month postoperative weight loss outcomes remained at one year following bariatric surgery. Further, in our recent publication, we proposed a potential clinical tool for precision medicine which predicted weight loss at six months post-RYGB based on liking ratings of the sweetened (10% added sucrose) half-and-half mixture. No predictive effect of this measure was found in VSG recipients [1]. Therefore, in the current study, we also determined if this preoperative clinical tool remained effective in predicting weight loss outcomes at one year following RYGB. Given that some patients experience weight regain after surgery, we measured changes in weight loss trajectories as defined by %TWL from six months to one year post-surgery (Δ%TWL) and assessed the relationship of these changes with our formerly identified preoperative predictors [1] as well as taste liking ratings and reported food cravings and dietary disinhibition as measured by the Food Craving Inventory (FCI) [13] and Three Factor Eating Questionnaire (TFEQ) [14], respectively.

## 2. Materials and Methods

The protocol was approved by the Johns Hopkins Institutional Review Board.

### 2.1. Participants

Adult women 18 to 55 years of age with a BMI ≥ 35 kg/m^2^ and approved for bariatric surgery at the Johns Hopkins Center for Bariatric Surgery were recruited and enrolled in this cohort study. Eligibility criteria and description of patient recruitment, enrollment, and participation are published in Smith et al., 2020 [1]. Each participant provided written informed consent at the initial visit. Participants completed five visits: 2 weeks ± 14 days before surgery (preoperative baseline), 2 weeks ± 14 days after surgery, 12 weeks ± 30 days after surgery, 24 weeks ± 30 days after surgery, and 52 weeks ± 30 days after surgery. Participant demographics were previously published [1]. In brief, participants did not differ in baseline characteristics (i.e., weight/BMI, race, age, baseline obesity comorbidities) with the exception that recipients of RYGB reported a higher prevalence of GERD at baseline relative to those receiving VSG.

### 2.2. Assessments

Participants completed a taste preference test and task-based fMRI, in which solutions were presented on the tongue via an MRI-compatible gustometer during functional scanning (see [1] for full description). The taste preference test always preceded the fMRI paradigm. For both assessments, participants were instructed to fast for at least 4 h prior to each study visit.

Demographic information was collected, and anthropometrics were measured using a high-capacity digital scale and stadiometer (see [1]). If a participant missed a research visit, body weight data were collected from electronic medical records (*n =* 1) or linear interpolation was used to calculate the missing value (*n =* 2). Participants completed a taste preference test modified from the version described in Drewnowski et al. [15]. In brief, using the “sip-and-spit” method, participants sampled 12 milk solutions with and without added sucrose from 30 cc cups and rated their preference on a digital 100-mm visual analogue scale (VAS) anchored by “not at all” (left anchor) to “very much” (right anchor). Qualtrics survey software was used to record preference ratings on the VAS. The 12 milk solutions were skim milk, whole milk, half-and-half, and cream prepared with 0%, 10%, or 20% added sugar and were presented in randomized order to each participant. For subsequent use in the fMRI paradigm, participants were also asked to select which of four solutions composed of varying concentrations of sodium bicarbonate and potassium chloride [16] was most perceived as a tasteless solution. A similar digital 100 mm VAS anchored by “not at all” (left anchor) to “very much” (right anchor) was used to collect hunger ratings (Qualtrics survey software).

Measures of taste-induced BOLD responses were acquired using a block design with pseudorandomized solution presentation modeled after Veldhuizen et al. [17] and scanning parameters previously detailed [1]. During stimulus delivery, participants were instructed to focus on the degree to which they liked or disliked the presented solution. The solution array delivered in the scanner was subject dependent. At baseline, the array consisted of a high-fat, no sugar solution (cream, 0% added sugar), a high sugar, no fat solution (skim milk, 20% added sugar), the tasteless solution, and the highest rated solution selected by the participant during the taste preference test. For postoperative follow-up visits, the array consisted of a high-fat, no sugar solution (cream, 0% added sugar), a high sugar, no fat solution (skim milk, 20% added sugar), the highest rated solution selected by the participant at baseline, the tasteless solution, and the highest rated solution selected by the participant during the taste preference test at the respective postoperative assessment. A water rinse followed delivery of each taste solution. Solutions were delivered at a rate of 0.4 mL/4 s and delivery was controlled using MATLAB (R2017b; MathWorks Inc., Portola Valley, CA, USA). See [1] for further description of stimulus delivery system and scanning parameters.

Self-reported food cravings and dietary disinhibition were assessed by the FCI [13] and TFEQ [14], respectively, at baseline, six months, and one year following bariatric surgery. The FCI is a validated self-report measure of total food cravings and cravings across four subscales, namely high fats, sweets, carbohydrates/starches, and fast-food fats. The TFEQ is a validated psychometric tool used to assess three dimensions of self-reported ingestive behavior—cognitive restraint, disinhibition, and hunger. For this study, the dietary disinhibition scale, which assesses one’s reported tendency to overeat in the presence of disinhibiting stimuli (e.g., palatable food), was administered and scored.

### 2.3. Data Analysis

Data were analyzed and graphed using GraphPad software (San Diego, CA, USA) and SPM12 (Wellcome Department of Cognitive Neurology, UCL, London, UK) with MATLAB software (MathWorks, Natick, MA, USA). For all statistical outcomes, the α level was established at 0.05 and post hoc comparisons were controlled for multiple comparisons when applicable.

Percent total weight loss was calculated using the following formula: ((preoperative or baseline weight) − (postoperative weight)) / ((preoperative or baseline weight)) × 100. Smaller values represent lower %TWL and larger values represent greater %TWL. Changes in %TWL from six months to one year (Δ%TWL) were calculated by subtracting %TWL observed at six months from %TWL observed at one year, with positive values representing weight gain and negative values representing weight loss. Paired-samples *t*-tests were performed to determine the effects of bariatric surgery type (surgery group) on %TWL, Δ%TWL, hunger ratings, taste preference ratings for sugar (taste mixtures collapsed across each fat concentration (skim, whole, half-and-half, cream)) and fat (taste mixtures collapsed across each sucrose concentration (0%, 10%, 20%)) at one year following bariatric surgery from baseline. Changes in reported food cravings and dietary disinhibition at six months and one year following bariatric surgery from baseline were assessed using repeated measures ANOVA. One-way repeated-measures ANOVAs were run to determine if VSG and RYGB altered taste preferences as a function of concentration (i.e., concentration-dependent responding) within each surgery group. To determine if our previously identified taste-related preoperative predictor of optimal weight loss success held true (i.e., recipients of RYGB who preferred the sugar-sweetened half-and-half mixture at baseline continued to lose more weight than those who received VSG regardless of baseline taste preference or RYGB recipients who did not report a preference for the mixture at baseline), a repeated measures ANOVA was run to compare %TWL, and Δ%TWL, across the following four designated groups at one year following bariatric surgery relative to baseline. Patients were grouped post hoc according to surgery received and bimodal split of the VAS; patients who rated the 10% sucrose-sweetened half-and-half mixture on the VAS with a 0–50 score at baseline were grouped as RYGB: non-preferers or VSG: non-preferers and those who rated the 10% sucrose-sweetened half-and-half mixture on the VAS with a 51–100 at baseline were grouped as RYGB: sweet-preferers or VSG: sweet-preferers.

Bivariate correlations of preoperative liking ratings and changes in taste preferences at 2 weeks from baseline (i.e., difference score) with %TWL at 12 months, as well as Δ%TWL, following surgery were conducted for each taste mixture to determine if the previously identified relationship of baseline taste preferences and acute changes therein were associated with improved weight loss outcomes at one year following bariatric surgery [1]. Difference scores were calculated by subtracting the 2-week postsurgical rating of each taste mixture from the pre-operative/baseline rating. To determine if self-reported food cravings and disinhibited eating prior to surgery were associated with weight loss outcomes, bivariate correlations of preoperative TFEQ dietary disinhibition and FCI scores with %TWL at six months and one year post-surgery were conducted. If significant relationships were identified, we then conducted bivariate correlations of changes in FCI and TFEQ dietary disinhibition scores with %TWL from baseline to six months and one year as well as ∆%TWL from six months to one year following surgery. We additionally correlated FCI and TFEQ dietary disinhibition scores at six months and one year post-surgery with %TWL at six months and one year post-surgery, respectively. Finally, to determine if food cravings and disinhibited eating contributed to ∆%TWL from six months to one year post-surgery, we conducted bivariate correlations of TFEQ dietary disinhibition and FCI scores at one year post-surgery with ∆%TWL at one year post-surgery, and correlated changes in TFEQ dietary disinhibition and FCI scores from baseline to one year post-surgery, and six months to one year post-surgery, with ∆%TWL.

Functional images were preprocessed as previously described [1]. Briefly, slice time correction, realignment to mean image, co-registration to the anatomical T1 image, segmentation, normalization to the Montreal Neurological Institute template [18], detrending, and smoothing using an 8 mm full-width, half-maximum isotropic Gaussian kernel were applied to the functional imaging data. The voxel size for functional images was 3 mm [3] and 1 mm [3] for structural images. Motion outliers were detected using the artifact detection tools toolbox for MATLAB (Gabrieli Laboratory, Massachusetts Institute of Technology, Cambridge, MA, USA) and motion parameters were included as regressors in the design matrix. Tastant-specific effects for the high fat, high sucrose, and baseline preferred test solutions and the tasteless solution were estimated using the general linear model and the BOLD response to the tasteless solution was subtracted from each test solution for analyses. We previously showed that baseline taste-induced BOLD responses in the VTA were associated with weight loss outcomes. Thus, here, the focus was isolated to the VTA. The VTA mask was adapted from the probabilistic atlas from Pauli et al. [19], and small-volume correction was applied and centered on the VTA. T-contrast maps thresholded at a peak level of *p* < 0.005 were used for displaying the results, and VTA activation was considered significant at a peak level of *p* < 0.05, family-wise error (FWE) corrected. Parameter estimates of the BOLD activation within the VTA at baseline and changes from baseline to 2 weeks post-surgery were extracted. Correlations were conducted to test associations between the extracted parameter estimates of BOLD activation within the VTA and changes in BOLD activation within the VTA at 2 weeks following surgery from baseline with %TWL at one year and Δ%TWL following RYGB and VSG separately.

## 3. Results

### 3.1. Percent Total Weight Loss at One Year Following Bariatric Surgery and Associations with Preoperative Taste Preferences

**Figure 1:**
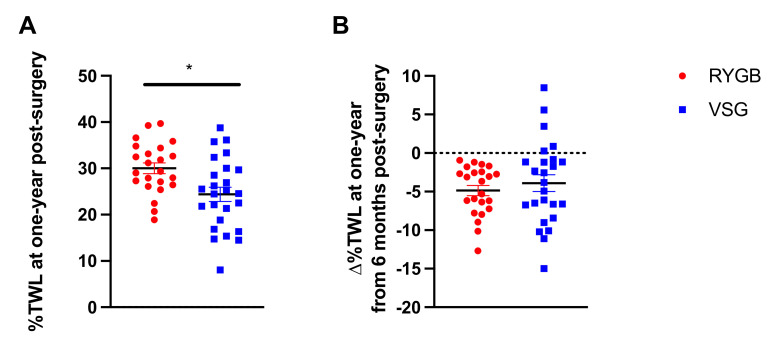
(**A**) Mean ± SEM (Standard Error of the Mean) %TWL (% Total Weight Loss) at one year following RYGB (Roux-en-Y Gastric Bypass; red circles) and VSG (Vertical Sleeve Gastrectomy; blue squares). (**B**) Mean ± SEM ∆%TWL from six months to one year following RYGB (red circles) and VSG (blue squares), where positive values indicate weight gain and negative values indicate weight loss. * *p* < 0.05.

**Figure 2:**
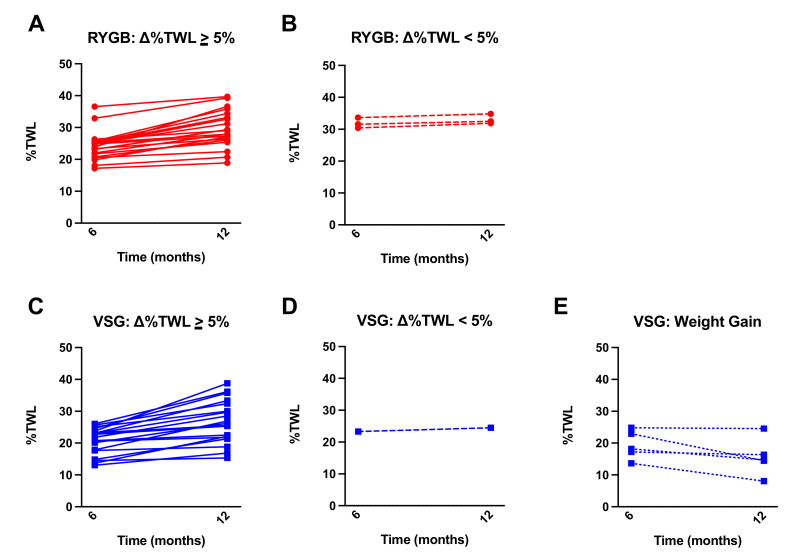
Mean ± SEM %TWL at six months and one year following surgery in patients who received (**A**) RYGB (red circles, solid lines) and showed equal to or greater than a 5% change in %TWL from six months to one year, (**B**) RYGB (red circles, dashed line) and showed less than a 5% change in %TWL from six months to one year, (**C**) VSG (blue squares, solid lines) and showed equal to or greater than a 5% change in %TWL from six months to one year, (**D**) VSG (blue squares, dashed lines) and showed less than a 5% change in %TWL from six months to one year, and (**E**) VSG (blue squares, dotted lines) and gained weight at 12 months relative to six months.

While both RYGB and VSG surgeries resulted in significant %TWL from baseline, patients who received RYGB (*n =* 23) had greater %TWL at one year following surgery compared with VSG (*n =* 25) (t(46) = 2.881, *p* = 0.006; Figure 1A). The change in %TWL from 6 months to one year was not statistically different between RYGB and VSG groups (t(46) = 0.740, *p* = 0.463; Figure 1B). Notably, a subset (*n =* 5, 20%) of the patients who received VSG, but none who received RYGB, experienced weight gain following their six-month assessment. Furthermore, the VSG group showed significantly greater variability in ∆%TWL relative to RYGB group (F(22,24) = 2.908, *p* = 0.014; Figure 1B). Figure 2 depicts surgery patients who showed greater than or equal to a 5% change in %TWL (Figure 2A,C), less than 5% change in %TWL (Figure 2B,D), and those who showed weight gain (Figure 2E) from six months to one year following RYGB and VSG. Percent TWL at six months and one year was positively correlated in both RYGB (*n =* 23; r = 0.819, *p* < 0.001) and VSG (*n =* 25; r = 0.732, *p* < 0.001). However, there was no association with %TWL at six months and Δ%TWL from six months to one year in either surgery group (VSG: r = −0.273, *p* = 0.188; RYGB: r = 0.140, *p* = 0.524).

**Figure 3:**
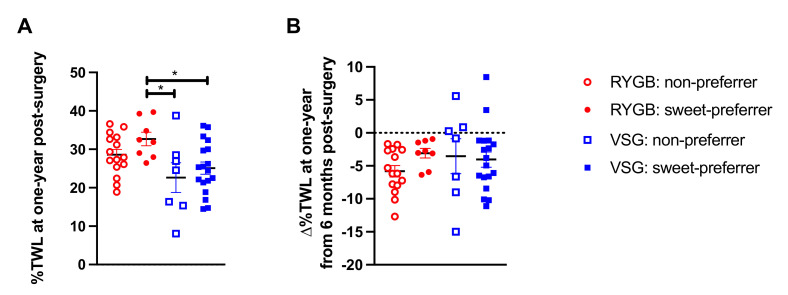
(**A**) Mean ± SEM %TWL at one year following surgery in patients who received RYGB (red circles) or VSG (blue squares) and rated the 10% sucrose-sweetened half-and-half mixture with a 0–50 score at baseline (open shapes) or rated the 10% sucrose-sweetened half-and-half mixture with a 51–100 score at baseline (closed shapes). (**B**) Mean ± SEM ∆%TWL from six months to one year following RYGB (red circles) and VSG (blue squares) and rated the 10% sucrose-sweetened half-and-half mixture with a 0–50 score at baseline (open shapes) or rated the 10% sucrose-sweetened half-and-half mixture with a 51–100 score at baseline (closed shapes), where positive values indicate weight gain and negative values indicate weight loss. * *p* < 0.05 Bonferroni-corrected comparison.

When we assessed the efficacy of our proposed preoperative clinical tool for predicting %TWL following bariatric surgery (i.e., baseline liking ratings for half-and-half with 10% added sucrose), a group difference was observed (F(3,44) = 3.677, *p* = 0.019; Figure 3A). Bonferroni-corrected post hoc analyses were conducted such that the mean of each VSG group (VSG: non-preferers and VSG: sweet-preferers) and the mean of the RYGB: non-preferers were compared with the mean of RYGB: sweet-preferers, the test group of interest that experienced greater %TWL at six months relative to all other groups [1]. RYGB: sweet-preferers continued to experience greater %TWL at one year following surgery relative to VSG: non-preferers (*p* = 0.018) and VSG: sweet-preferers (*p* = 0.032), but the comparison with RYGB: non-preferers was no longer significant (*p* = 0.510). No differences in %TWL at one year post-surgery were observed if post hoc analyses were restricted to RYGB: sweet-preferers and RYGB: non-preferers. Analyses were also conducted on ∆%TWL from six months to one year between the surgery groups and revealed no difference as a function of baseline sweet preference status in either patients who received RYGB or VSG (F(3,44) = 0.836, *p* = 0.481; Figure 3B). If analyses were restricted to the RYGB group, RYGB: non-preferers showed greater ∆%TWL from six months to one year relative to RYGB: sweet-preferers (*p* = 0.049).

Unlike the observed relationship between preoperative liking ratings for sugar-sweetened mixtures (i.e., skim milk and whole milk with 20% added sucrose, half-and-half with 10% added sucrose) and %TWL at six months post-RYGB [1], preoperative liking ratings for the mixtures were no longer significantly associated with %TWL in RYGB (*p*-values ≥ 0.073), or VSG (*p*-values ≥ 0.150), at one year follow-up. No associations were found between ∆%TWL and preoperative liking ratings (*p*-values ≥ 0.073).

### 3.2. Changes in Liking Ratings for Sucrose-Containing Mixtures and Fat-Containing Mixtures One-Year Following Bariatric Surgery

**Figure 4:**
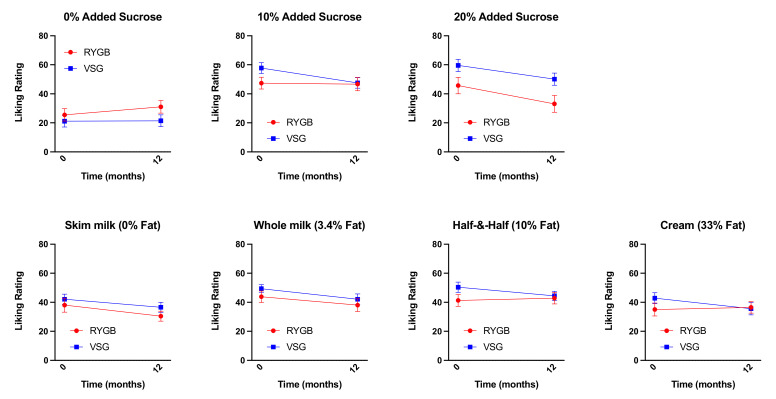
Mean ± SEM liking ratings for the mixtures varying in sucrose content (0%, 10%, and 20%) collapsed across all fat content and mixtures varying in fat content (0%, 3.4%, 10%, and 33%) collapsed across all sucrose concentrations at baseline (0 weeks) and one year following RYGB (red circles) and VSG (blue squares).

We previously showed that patients, prior to bariatric surgery (regardless of surgery group), reported greater liking for sucrose-sweetened mixtures compared with mixtures without added sugar [1]. No differences in liking ratings of mixtures varying in fat content were identified at baseline or at six months post-surgery [1]. However, following RYGB, patients reported similar liking for all mixtures regardless of sucrose content, driven by a decrease in liking ratings for the mixtures prepared with added sucrose by three months and up to six months post-surgery [1]. Patients who received VSG continued to like sucrose-sweetened mixtures more than mixtures without added sugar [1]. Paired sample *t*-tests showed no differences between baseline and one year post-surgery liking ratings for mixtures varying in fat or sugar concentration in either the RYGB or VSG group, although decreases in liking ratings for 20% added sugar (*p* = 0.055) in RYGB patients and decreases in liking ratings for 10% added sugar (*p* = 0.058) in the VSG patients trended toward significance (Figure 4). Importantly, there were no differences between six-month and one-year liking ratings for mixtures varying in fat or sugar concentration following RYGB or VSG (*p*-values ≥ 0.155), suggesting that the decreases in liking ratings at six months following RYGB identified previously [1] are somewhat maintained at one year.

### 3.3. Changes in Liking Ratings at 2 Weeks Following RYGB, but Not VSG, Were Associated with One-Year Weight Loss

**Figure 5:**
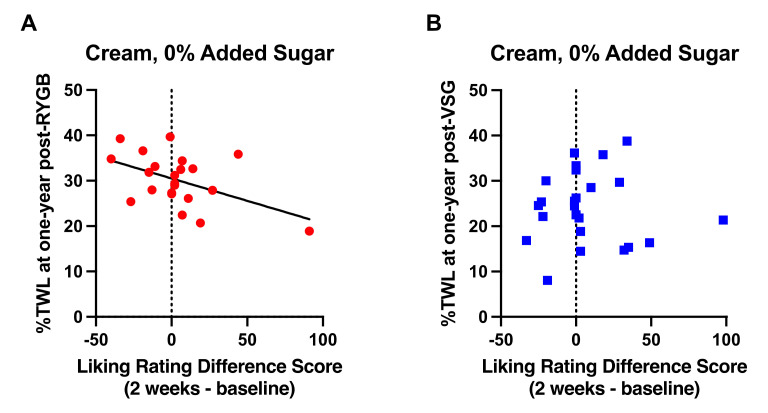
Relationship of difference scores demonstrating changes in liking ratings for cream with no added sucrose at 2 weeks after surgery from baseline with %TWL at one year in patients who received (**A**) RYGB (red circles) and (**B**) VSG (blue squares). Line of best fit represents significant correlation.

As observed at six months following RYGB [1], postoperative changes in liking ratings for the high fat mixture (cream, 0% added sucrose) that occurred 2 weeks following surgery relative to baseline negatively correlated with %TWL at one year following RYGB (*n =* 22; r = −0.486, *p* = 0.022, Figure 5A); patients who provided a lower rating (greater dislike) for the high fat mixture at 2 weeks post-RYGB relative to baseline experienced more %TWL than those whose ratings increased following RYGB. There were no associations between postoperative changes in taste ratings and %TWL following VSG (*n =* 24; r = −0.002, *p* > 0.992; Figure 5B) or Δ%TWL from six months to one year post-surgery in either surgery group (RYGB: r = 0.080, *p* = 0.725; VGS: r = −0.131, *p* = 0.543).

### 3.4. Association of Taste-Induced BOLD Signaling in the VTA with Weight loss at One Year Following Bariatric Surgery

We previously showed preoperative BOLD responses in the VTA to the high fat (cream, 0% added sucrose), high sugar (skim milk, 20% added sucrose), and preoperative preferred mixtures to be negatively correlated with %TWL at six months following RYGB but not VSG [1]. At one year follow-up, preoperative BOLD signaling in the VTA in response to the high fat (r = −0.401, *p* = 0.089), high sugar (r = −0.378, *p* = 0.111), and most preferred (r = −0.210, *p* = 0.388) mixtures was no longer associated with %TWL in the RYGB group (*n =* 19). Neither was there an association of the VTA BOLD response to the high fat (r = −0.088, *p* = 0.711), high sugar (r = −0.244, *p* = 0.300), and most preferred (r = 0.049, *p* = 0.837) mixtures with %TWL in the VSG group (*n* = 20). Similarly, changes in VTA BOLD signaling at 2 weeks from baseline in response to these mixtures did not correlate with %TWL one year after RYGB (*n =* 15; *p*-values ≥ 0.173) or VSG (*n* = 17; *p*-values ≥ 0.611).

**Figure 6:**
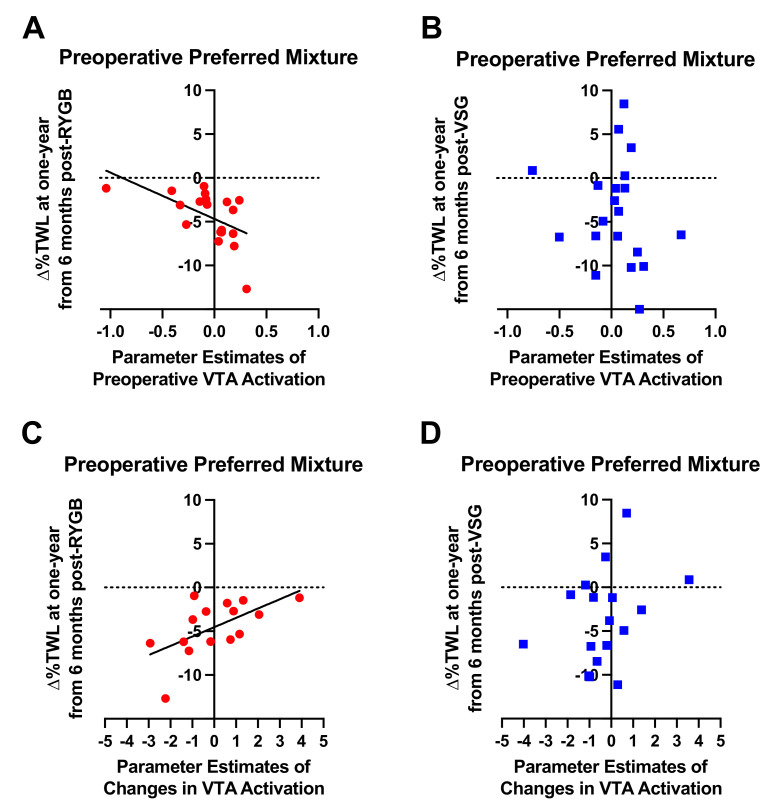
Relationship between preoperative VTA activation in response to the patient-specific preferred mixture delivered in the MRI scanner and ∆%TWL from six months to one year in patients who received (**A**) RYGB (red circles) and (**B**) VSG (blue squares). Relationship between changes in preoperative VTA activation at 2 weeks following surgery relative to baseline in response to the patient-specific preferred mixture delivered in the MRI scanner and ∆%TWL from six months to one year in patients who received (**C**) RYGB (red circles) and (**D**) VSG (blue squares). Line of best fit represents significant correlation.

Preoperative VTA BOLD activation to the preoperative preferred mixture, but not high fat (r = −0.362, *p* = 0.128) or high sugar (r = −0.395, *p* = 0.095) was associated with Δ%TWL from six months to one year following RYGB (*n =* 19; r = −0.553, *p* = 0.014; Figure 6A). RYGB patients who showed less VTA BOLD activation at baseline in response to the preoperative preferred mixture experienced less weight loss from six months to one year after RYGB. No associations of ∆%TWL with preoperative VTA BOLD activation in response to the taste mixtures were detected in the VSG group (*n* = 20; *p*-values ≥ 0.481, Figure 6B). Similar with preoperative VTA activation in the RYGB group, changes in VTA BOLD signaling at 2 weeks from baseline in response to the preoperative preferred mixture were positively associated with Δ%TWL one year after RYGB (*n* = 15; r = 0.600, *p* = 0.018; Figure 6C). Patients who showed increases in VTA BOLD activation at 2 weeks from baseline in response to the preoperative preferred mixture showed less weight loss from six months to one year after RYGB.

### 3.5. Self-Reported Food Craving and Eating Disinhibition at Six Months and One Year Following Bariatric Surgery from Baseline and Relationships with Weight Loss Outcomes

**Figure 7:**
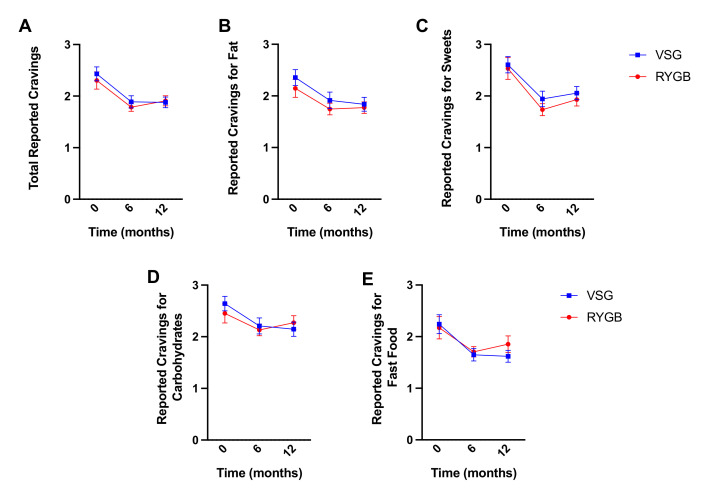
Mean ± SEM self-reported scores on the FCI for (**A**) total cravings, (**B**) fat cravings, (**C**) sweet cravings, (**D**) carbohydrates/starch cravings, and (**E**) fast food cravings at baseline, six months, and one year following RYGB (red circles) and VSG (blue squares).

Self-reported total food cravings decreased following bariatric surgery (RYGB: N = 18, VSG: *n* = 21; F(2,74) = 25.114, *p* < 0.001) but did not differ between groups (F(1,37) = 0.330, *p* = 0.569) nor was there a surgery group x time interaction (F(2,74) = 0.344, *p* = 0.710) (Figure 7A). Similar main effects of time were observed for cravings for high fat (F(2,74) = 9.516, *p* < 0.001; Figure 7B), sweets (F(2,74) = 22.845, *p* < 0.001; Figure 6C), carbohydrates/starches (F(2,74) = 10.552, *p* < 0.001; Figure 7D), or fast food fats (F(2,74) = 8.299, *p* = 0.001; Figure 7E). Bonferroni-corrected pairwise comparisons revealed significant decreases in food cravings at six months and one year following surgery relative to baseline for total (6m: *p* < 0.001, 1y: *p* < 0.001), high fat (6m: *p* = 0.003, 1y: *p* = 0.006), sweets (6m: *p* < 0.001, 1y: *p* < 0.001), carbohydrates/starches (6m: *p* = 0.002, 1y: *p* = 0.003), and fast food (6m: *p* = 0.004, 1y: *p* = 0.018), but no differences in cravings across the domains reported at 6 months and one year following surgery (*p*-values ≥ 0.570). No surgery group main effect (*p*-values ≥ 0.464) or interaction with time (*p*-values ≥ 0.260) was observed for any of the subscales.

**Figure 8:**
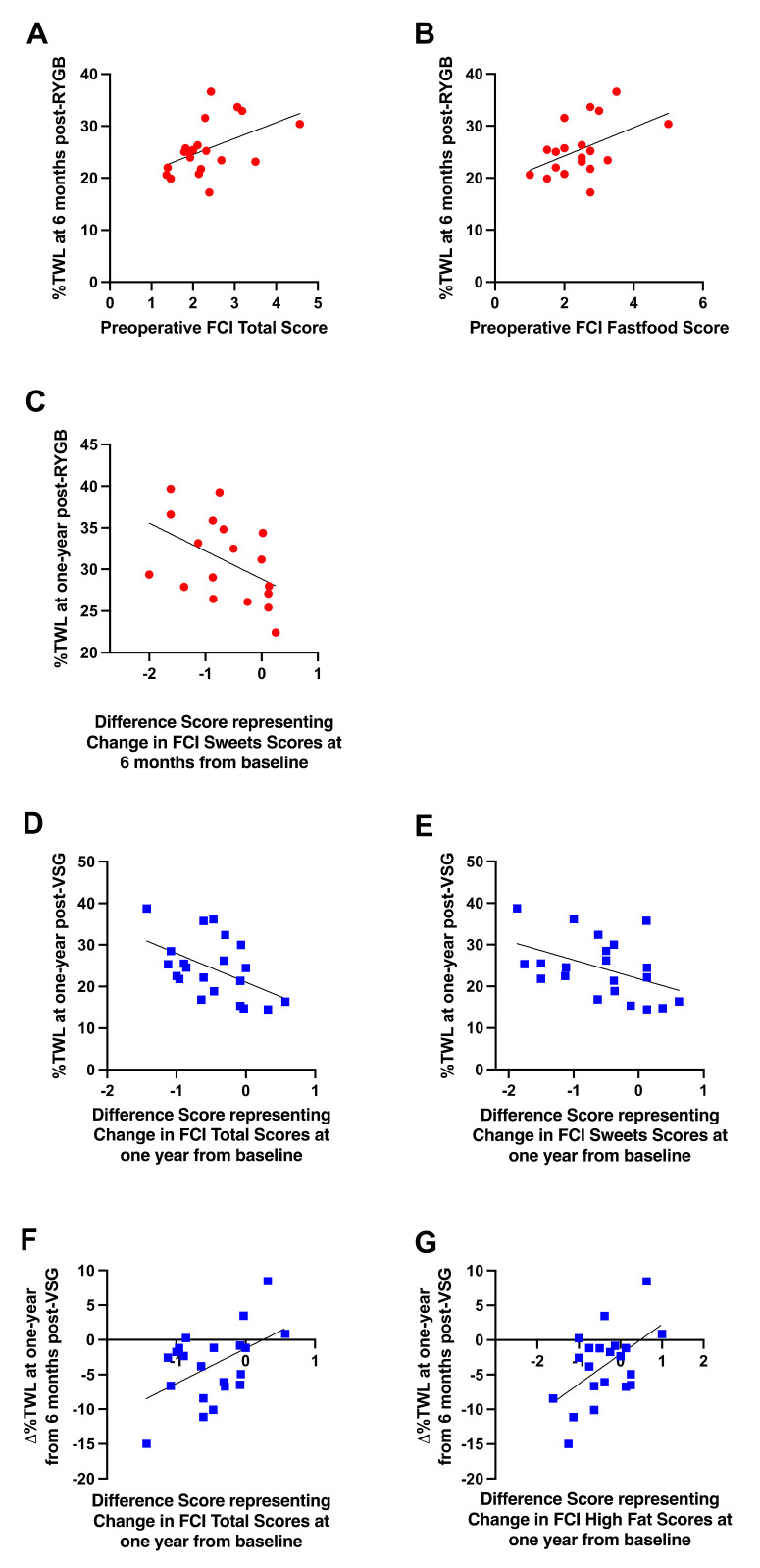
Significant relationships between (**A**) preoperative self-reported total craving scores and %TWL at six months post-RYGB (red circles), (**B**) preoperative self-reported cravings for fast food and %TWL at six months post-RYGB (red circles), (**C**) changes in self-reported sweet cravings at six months from baseline and %TWL at one year post-RYGB (red circles), (**D**) changes in self-reported total food cravings at one year from baseline and %TWL at one year post-VSG (blue squares), (**E**) changes in self-reported sweet cravings at one year from baseline and %TWL at one year post-VSG (blue squares), (**F**) changes in self-reported total food cravings at one year from baseline and ∆%TWL from six months to one year post-VSG (blue squares), (**G**) changes in self-reported fat cravings at one year from baseline and ∆%TWL from six months to one year post-VSG (blue squares). Line of best fit represents significant correlation.

Preoperative total food cravings (r = 0.470, *p* = 0.037; Figure 8A) and cravings for fast food (r = 0.469, *p* = 0.037; Figure 8B) were positively associated with %TWL at six months, but not one year, following RYGB; greater cravings prior to RYGB were associated with higher %TWL at six months. Following surgery, changes in sweet cravings at six months post-RYGB from baseline were negatively correlated with %TWL at one year (r = −0.470, *p* = 0.049; Figure 8C); patients who received RYGB and reported greater decreases in cravings for sweets at six months after surgery relative to baseline experienced more %TWL at one year than those who reported smaller decreases or increases in sweet cravings.

No associations with preoperative FCI scores and body weight outcomes were detected in patients who received VSG. Following VSG, changes in self-reported total cravings (r = −0.493, *p* = 0.023; Figure 8D) and sweet cravings (r = −0.447, *p* = 0.042; Figure 8E) at one year post-surgery from baseline were negatively correlated with %TWL at one year; patients who received VSG and reported greater decreases in total food cravings as well as sweet cravings at one year after surgery relative to baseline experienced more %TWL at one year than those who reported smaller decreases or increases in cravings. Further, changes in total food cravings (r = 0.495, *p* = 0.023; Figure 8F) and high fat cravings (r = 0.535, *p* = 0.012; Figure 8G) at one year post-VSG relative to baseline positively correlated with ∆%TWL at one year relative to six months; patients who received VSG and reported greater decreases in total food cravings and cravings for fat at one year after surgery relative to baseline continued to lose weight from six months to one year, whereas those who reported smaller decreases or increases in cravings at one year post-VSG relative to baseline exhibited reduced weight loss or weight gain from six months to one year.

### 3.6. Three Factor Eating Questionnaire

**Figure 9:**
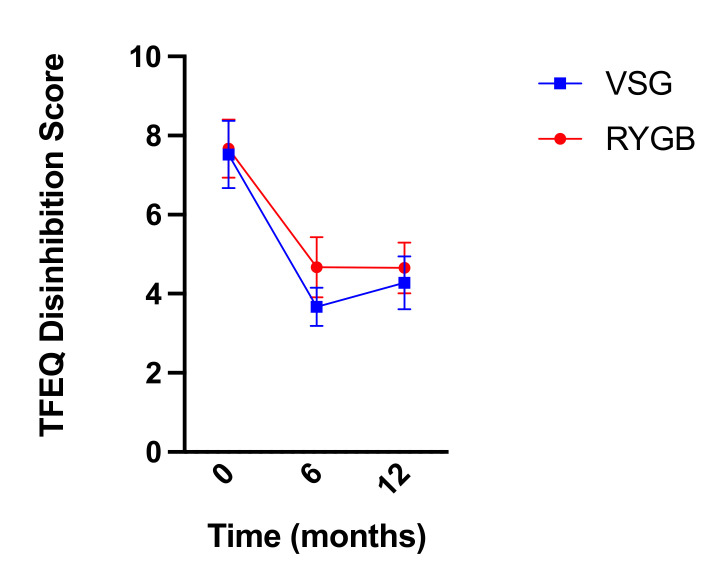
Mean ± SEM self-reported scores on the TFEQ disinhibition scale at baseline, six months, and one year following RYGB (red circles) and VSG (blue squares).

Similarly, self-reported dietary disinhibition as measured by the TFEQ decreased following bariatric surgery (RYGB: *n =* 18, VSG: *n =* 21; F(2,74) = 30.586, *p* < 0.001; Figure 9) but did not differ between groups (F(1,37) = 1.475, *p* = 0.232) nor was there a surgery group x time interaction (F(2,74) = 0.802, *p* = 0.452). Bonferroni-corrected pairwise comparisons revealed significant decreases in dietary disinhibition from baseline to six months post-surgery and baseline to one year post-surgery (*p*-values < 0.001). Dietary disinhibition did not differ between reports at six months post-surgery and reports at one year post-surgery (*p* = 1.00) in either group.

**Figure 10:**
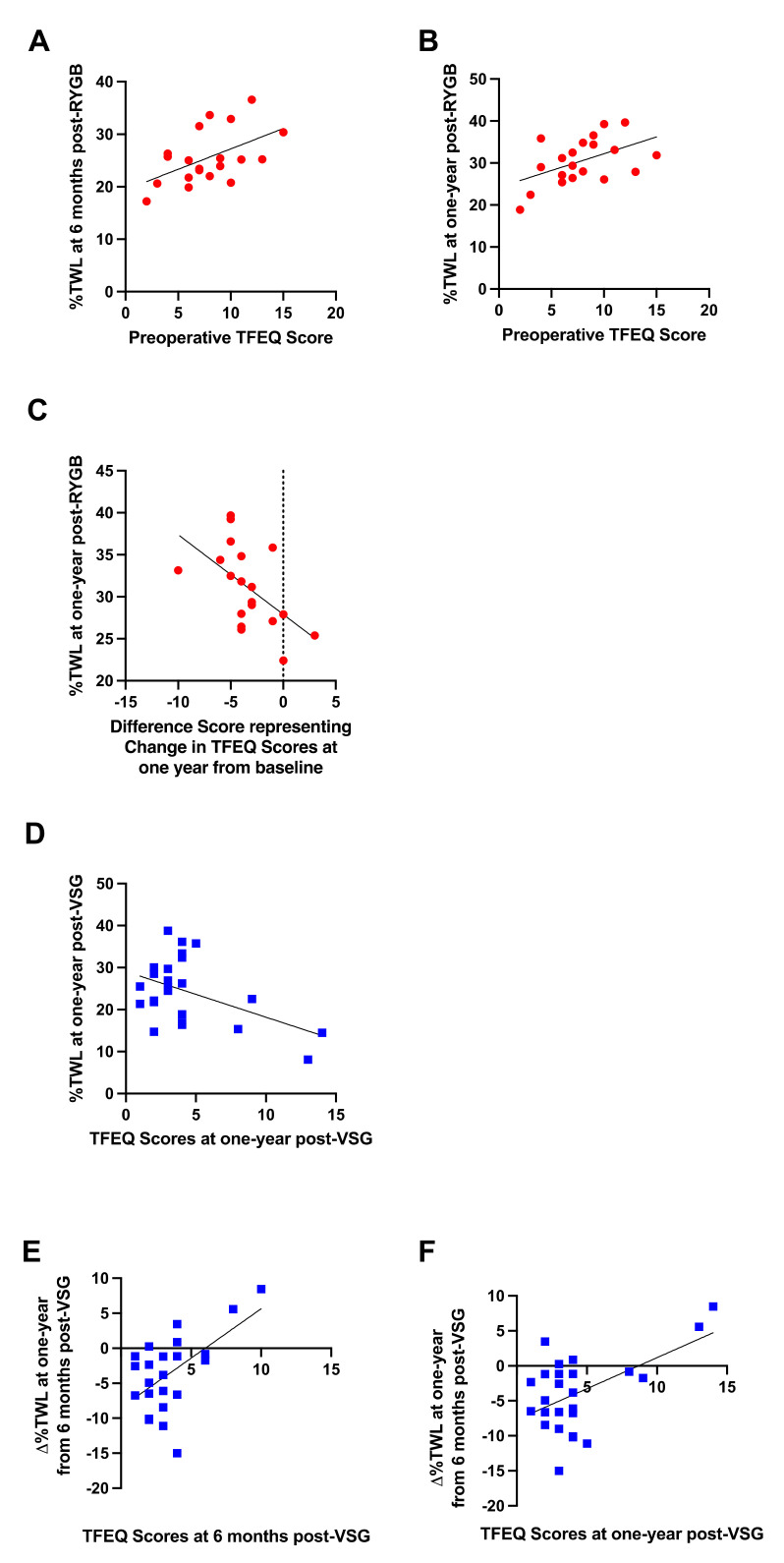
Significant relationships between (**A**) preoperative self-reported dietary disinhibition and %TWL at six months post-RYGB (red circles), (**B**) preoperative self-reported dietary disinhibition and %TWL at one year post-RYGB (red circles), (**C**) changes in self-reported dietary disinhibition at one year from baseline and %TWL at one year post-RYGB (red circles), (**D**) self-reported dietary disinhibition at one year from baseline and %TWL at one year post-VSG (blue squares), (**E**) self-reported dietary disinhibition at six months post-VSG and ∆%TWL from six months to one year post-VSG (blue squares), (**F**) self-reported dietary disinhibition at one year post-VSG and ∆%TWL from six months to one year post-VSG (blue squares). Line of best fit represents significant correlation.

Preoperative dietary disinhibition as measured by the TFEQ positively correlated with %TWL at six months (r = 0.516, *p* = 0.020; Figure 10A) and one year (r = 0.495, *p* = 0.026; Figure 10B) post-RYGB; greater dietary disinhibition reported prior to surgery was associated with better %TWL post-RYGB. The change in dietary disinhibition at one year post-RYGB relative to baseline negatively correlated with %TWL at one year following surgery (r = −0.543, *p* = 0.016; Figure 10C); decreases in dietary disinhibition following surgery were associated with greater %TWL at one year post-RYGB.

No associations with preoperative dietary disinhibition scores and body weight outcomes were detected in patients who received VSG. Following VSG, dietary disinhibition at one year post-surgery negatively correlated with %TWL at one year (r = −0.470, *p* = 0.018; Figure 10D); lower dietary disinhibition at one year post-VSG was associated with better %TWL. Further, both dietary disinhibition at six months (r = 0.568, *p* = 0.005; Figure 10E) and one year (r = 0.541, *p* = 0.005; Figure 10F) post-VSG positively correlated with ∆%TWL from six months to one year; higher dietary disinhibition scores at six months and one year following VSG were associated with poorer weight loss or weight gain from six months to one year after surgery.

## 4. Discussion

In this observational study, we followed patients up to one year following RYGB and VSG to determine if previously identified behavioral and brain-related taste-associated predictors of weight loss at six months post-surgery, particularly in RYGB patients, persisted. Previously, we reported that greater preoperative liking of sugar-sweetened mixtures was associated with better six-month weight loss outcomes among RYGB patients [1]. As measured by functional neuroimaging, lower preoperative VTA activation and increased 2-week changes in VTA responses to sugar- and fat-containing mixtures also correlated with increased weight loss after RYGB, but not VSG [1]. We hypothesized that anatomical and metabolic changes resulting from RYGB reset the neural processing of reward stimuli in the mesolimbic pathway [1], at least up to six months post-surgery. The self-reported food cravings and dietary disinhibition data collected here were consistent with these findings. Participants who reported higher food cravings and dietary disinhibition and then underwent RYGB, but not VSG, showed better %TWL at six months than those who had lower preoperative food cravings and then underwent RYGB surgery, supporting the hypothesis that RYGB modifies the mesolimbic dopamine system.

At one year follow-up, the previously identified relationships between %TWL at six months and baseline liking ratings of sugar-sweetened mixtures and BOLD responses to sugar- and fat-containing mixtures were not present [1]. One possible contributor to the lack of association at the one-year time point may be that patients reverted to their former, preoperative obesogenic eating behaviors. Studies using an ad libitum buffet meal to measure food preferences (selection) and intake revealed no changes in food choice at six months and 18 months following RYGB or VSG relative to baseline [20,21]. In rodent models, continued exposure to energy-dense foods results in blunted responsivity in brain reward circuits [22,23,24,25]. Similarly, in individuals with obesity, chronic consumption of foods high in fat and sugar correlates with lower BOLD-signaling in brain reward circuits [26] and lower levels of striatal D2R availability [27,28,29]. Although we did not directly assess food intake in the current study, the decreased weight loss and changes in food cravings and disinhibition that we observed are consistent with a gradual increase in palatable food intake after a post-surgical nadir. Thus, a return to preoperative eating practices in patients who have undergone bariatric surgery may negate or reverse the benefits that RYGB appears to have on neural processing of reward stimuli in the mesolimbic pathway [1,30,31]. An alternative but not mutually exclusive hypothesis may be that the superior weight loss experienced in the initial six months of surgery by patients who showed more obesogenic phenotypes (i.e., lower VTA activation in response to high sugar and high fat containing mixtures, greater liking for sucrose-sweetened mixtures) prior to RYGB began to stabilize at the one-year time point. A change in the slope of the weight loss curve in RYGB patients, particularly those who had greater obesogenic phenotypes prior to surgery, may also explain why patients who showed greater VTA BOLD activation at baseline and increases therein at 2 weeks from baseline in response to the preoperative preferred mixture showed less weight loss from six months to one year after RYGB (Figure 6A,C). It is also possible that changes to the reward system caused by surgical disruption of the gut–brain axis have a time-limited effect due to non-dietary mechanisms causing resistance of the system to change (e.g., a reversion to genetically driven tendencies), or that different brain regions within a broader reward system become more influential beyond six months post-surgery. Complementary mechanistic studies in pre-clinical models with controlled dietary intake pre- and post-surgery would be needed to more fully test independent effects of diet and surgery on taste-related reward. Regardless of the underlying mechanisms, these data nevertheless suggest that six months may be a critical time for mitigating weight gain or maintaining successful weight loss trajectories following bariatric surgery.

We previously demonstrated that while initial weight loss outcomes (within 2 weeks of surgery) were similar between RYGB and VSG, those who received RYGB experienced significantly greater weight loss relative to those who received VSG by six months post-surgery [1]. Data presented here demonstrate that RYGB remained more effective for weight loss than VSG up to one year, consistent with the previous literature. As observed in our previous report at six months post-surgery, no changes in reported hunger from baseline to one year following surgery were identified (Time: F(1,42) = 0.002, *p* = 0.965; Surgery Group: F(1,42) = 0.190, *p* = 0.665; Time x Surgery Group: F(1,42) = 0.621, *p* = 0.435), suggesting that perceived hunger is not a driver of differences in weight observed between the two surgical groups. A possible mechanism responsible for improved weight loss in RYGB patients could be due to postoperative changes in taste preference for fat. Acute changes in taste preference occurring at the onset of the dynamic weight loss phase, when weight loss results primarily from profound negative energy balance, appear to remain a factor or have continuing consequences on factors that influence weight loss in patients who receive RYGB (Figure 5). While a preoperative feature or psychometric instrument for determining which individuals will show reduced liking for fat following RYGB has not been identified, implementing early post-operative weight management strategies in patients who show null or increased preferences for fat following surgery, and thus may continue to consume a diet higher in fat postoperatively and be at higher risk for weight recidivism, may facilitate improved weight loss outcomes in these patients. Self-reported data collected here support the need for such interventions. Patients who reported greater decreases in food cravings and dietary disinhibition at one year after VSG relative to baseline showed better weight loss at one year following surgery and had better weight loss trajectories from six months to one year relative to those who reported smaller decreases or increases in cravings (Figure 8D–G) and dietary disinhibition at one year post-VSG (Figure 10D–F). Isolation of this effect to the VSG group may be due to the greater variability and weight regain observed in the VSG, but not RYGB, cohort.

## 5. Conclusions

Personalization of bariatric procedures for optimal weight loss and alleviation of obesity-related comorbidities in patients with obesity is critical. However, if behaviors that facilitate the maintenance of successful weight loss are not continued, then the identification of such predictors for personalized medicine loses their value. Here, we show that the resetting of the neural processing of reward stimuli in the mesolimbic pathway following RYGB [1] may be temporary and is likely contingent upon post-operative eating behaviors. Patients who continue to consume foods high in sugar and fat, particularly during the weight maintenance phase after undergoing bariatric surgery, may lose the benefit that RYGB has on brain reward. The short-term restoration of VTA responsivity (within 2 weeks of surgery) potentially responsible for postoperative shifts in dietary choices toward less energy-dense foods [32,33,34,35,36] may be reversed if the healthier diet is not maintained. These data have significant practical implications for clinicians involved in the care of preoperative and postoperative bariatric patients, including a critical window of vulnerability to consuming high calorie foods leading to weight gain that should be addressed for optimal and maintained weight loss success. Studies investigating whether implementing additional interventions and resources at six months post-surgery resulting in improved weight loss outcomes are thus warranted.

## Data Availability

The data presented in this study are available on request from the corresponding author.
